# Diversity, ecology and intestinal function of bifidobacteria

**DOI:** 10.1186/1475-2859-13-S1-S4

**Published:** 2014-08-29

**Authors:** Francesca Bottacini, Marco Ventura, Douwe van Sinderen, Mary O'Connell Motherway

**Affiliations:** 1 Alimentary Pharmabiotic Centre and School of Microbiology, National University of Ireland, Cork, Western Road, Cork, Ireland; 2 Laboratory of Probiogenomics, Department of Life Sciences, University of Parma, Italy

## Abstract

The human gastrointestinal tract represents an environment which is a densely populated home for a microbiota that has evolved to positively contribute to host health. At birth the essentially sterile gastrointestinal tract (GIT) is rapidly colonized by microorganisms that originate from the mother and the surrounding environment. Within a short timeframe a microbiota establishes within the (breastfed) infant's GIT where bifidobacteria are among the dominant members, although their numerical dominance disappears following weaning. The numerous health benefits associated with bifidobacteria, and the consequent commercial relevance resulting from their incorporation into functional foods, has led to intensified research aimed at the molecular understanding of claimed probiotic attributes of this genus. In this review we provide the current status on the diversity and ecology of bifidobacteria. In addition, we will discuss the molecular mechanisms that allow this intriguing group of bacteria to colonize and persist in the GIT, so as to facilitate interaction with its host.

## Introduction

Bifidobacteria are typical gut inhabitants, and represent non-motile, non-sporulating, non-gas producing, saccharolytic Gram-positive bacteria that belong to the family *Bifidobacteriaceae *and the phylum *Actinobacteria*, the latter being one of the largest and most populated taxonomic units in the domain *Bacteria*, and being composed of six classes and 16 orders [1]. *Actinobacteria *exhibit a wide spectrum of morphologies and metabolic properties, and possess genomes of a high G+C content, ranging from 46% to over 70% (for example members of the *Corynebacterium *genus) [1]. Furthermore, certain representatives of this phylum produce a range of important secondary metabolites, including those that are exploited as antibiotics by the pharmaceutical industry (produced by *Streptomyces *spp.) [2,3].

During the last two to three decades bifidobacteria have become the subject of intensifying scientific scrutiny because they represent an abundant bacterial component of the human GIT microbiota, while they are believed to be the most dominant bacterial group present in the gut microbiota of vaginally delivered, breast-fed infants [4]. They also are known to stably colonize the GIT of various other eukaryotic hosts, including mammals, birds and insects [5,6]. Their discovery has been attributed to Henri Tissier who in 1899 isolated a so-named *Bacillus bifidus *from breast-fed infant faeces [7]; bifidobacteria were incorrectly assigned to the genus *Lactobacillus *for much of the 20^th ^century, and only relatively recently obtained appropriate classification as a separate genus, *Bifidobacterium *[8].

## Taxonomic diversity and ecology

### The *Bifidobacterium *genus

The *Bifidobacterium *genus currently comprises 48 recognized species (Table 1) [9-13]. According to taxonomic classification based on comparative analyses of 16S rRNA-encoding DNA and concatenated multilocus sequences, representing a number of conserved housekeeping genes (*clpC, dnaJ, xfp, dnaB, rpoC *and *purF*), bifidobacterial species have been clustered into six main phylogenetic clusters, consisting of the *Bifidobacterium boum, Bifidobacterium asteroides, Bifidobacterium adolescentis, Bifidobacterium pullorum, Bifidobacterium longum*, and *Bifidobacterium pseudolongum *phylogenetic groups, where *Bifidobacterium asteroides*, isolated from the insect gut, appears to be the closest relative of the ancient progenitor of the genus *Bifidobacterium *[9].

**Table 1:** List of currently recognized bifidobacterial species with their isolation sources.

Species	Type strain	Origin	Sequencing status
***Bifidobacterium actinocoloniiforme***	DSM 22766	Bumblebee digestive tract	-
***Bifidobacterium adolescentis***	ATCC 15705	Adult faeces	COMPLETE
***Bifidobacterium angulatum***	ATCC 27535	Adult faeces	DRAFT
***Bifidobacterium animalis *subsp. *animalis***	ATCC 25527	Sewage	COMPLETE
***Bifidobacterium animalis *subsp. *lactis***	DSM 10140	Fermented milk	COMPLETE
***Bifidobacterium asteroides***	ATCC 25910	Bee intestine	COMPLETE
***Bifidobacterium biavatii***	DSM 23969	Tamarind faeces	-
***Bifidobacterium bifidum***	ATCC 29521	Infant faeces	COMPLETE
***Bifidobacterium bohemicum***	DSM 22767	Bumblebee digestive tract	-
***Bifidobacterium bombi***	DSM 19703	Bumblebee digestive tract	-
***Bifidobacterium boum***	ATCC 27917	Bovine rumen	-
***Bifidobacterium breve***	ATCC 15700	Infant faeces	COMPLETE
***Bifidobacterium callitrichos***	DSM 23973	Marmoset faeces	-
***Bifidobacterium catenulatum***	ATCC 27539	Adult faeces	DRAFT
***Bifidobacterium choerinum***	ATCC 27686	Piglet faeces	-
***Bifidobacterium coryneforme***	ATCC 25911	Bee intestine	-
***Bifidobacterium crudilactis***	LMG 23609	Raw milk cheese	-
***Bifidobacterium cuniculi***	ATCC 27916	Rabbit faeces	-
***Bifidobacterium dentium***	ATCC 27534	Oral cavity	COMPLETE
***Bifidobacterium gallicum***	ATCC 49850	Human faeces	DRAFT
***Bifidobacterium gallinarum***	ATCC 33777	Chicken caecum	-
***Bifidobacterium indicum***	ATCC 25912	Bee intestine	-
***Bifidobacterium kashiwanohense***	DSM 21854	Infant faeces	-
***Bifidobacterium longum *subsp. *infantis***	ATCC 15697	Infant faeces	COMPLETE
***Bifidobacterium longum *subsp. *longum***	ATCC 15707	Adult faeces	COMPLETE
***Bifidobacterium longum *subsp. *suis***	ATCC 27533	Piglet faeces	-
***Bifidobacterium magnum***	ATCC 27540	Rabbit faeces	-
***Bifidobacterium merycicum***	ATCC 49391	Bovine rumen	-
***Bifidobacterium minimum***	ATCC 27538	Sewage	-
***Bifidobacterium mongoliense***	DSM 21395	Fermented milk	-
***Bifidobacterium moukalabense***	JCM 18751	Gorilla faeces	-
***Bifidobacterium pseudocatenulatum***	ATCC 27919	Infant faeces	DRAFT
***Bifidobacterium pseudolongum *subsp. *globosum***	ATCC 25865	Bovine rumen	-
***Bifidobacterium pseudolongum *subsp. *pseudolongum***	ATCC 25526	Pig faeces	-
***Bifidobacterium psychraerophilum***	LMG 21775	Porcine caecum	-
***Bifidobacterium pullorum***	ATCC 27685	Chicken faeces	-
***Bifidobacterium reuteri***	DSM 23975	Marmoset faeces	-
***Bifidobacterium ruminantium***	ATCC 49390	Bovine rumen	-
***Bifidobacterium saeculare***	ATCC 49392	Rabbit faeces	-
***Bifidobacterium sanguini***	DSM 23967	Tamarind faeces	-
***Bifidobacterium scardovii***	DSM 13734	Human sources	-
***Bifidobacterium stellenboschense***	DSM 23968	Tamarind faeces	-
***Bifidobacterium stercoris***	JCM 15918	Adult faeces	-
***Bifidobacterium subtile***	ATCC 27537	Sewage	-
***Bifidobacterium thermacidophilum *subsp. *porcinum***	DSM 17755	Piglet faeces	-
***Bifidobacterium thermacidophilum *subsp. *thermoacidophilum***	DSM 15837	Anaerobic digester	-
***Bifidobacterium thermophilum***	ATCC 25525	Piglet faeces	COMPLETE
***Bifidobacterium tsurumiense***	JCM 13495	Hamster dental plaque	-

**Figure 1:**
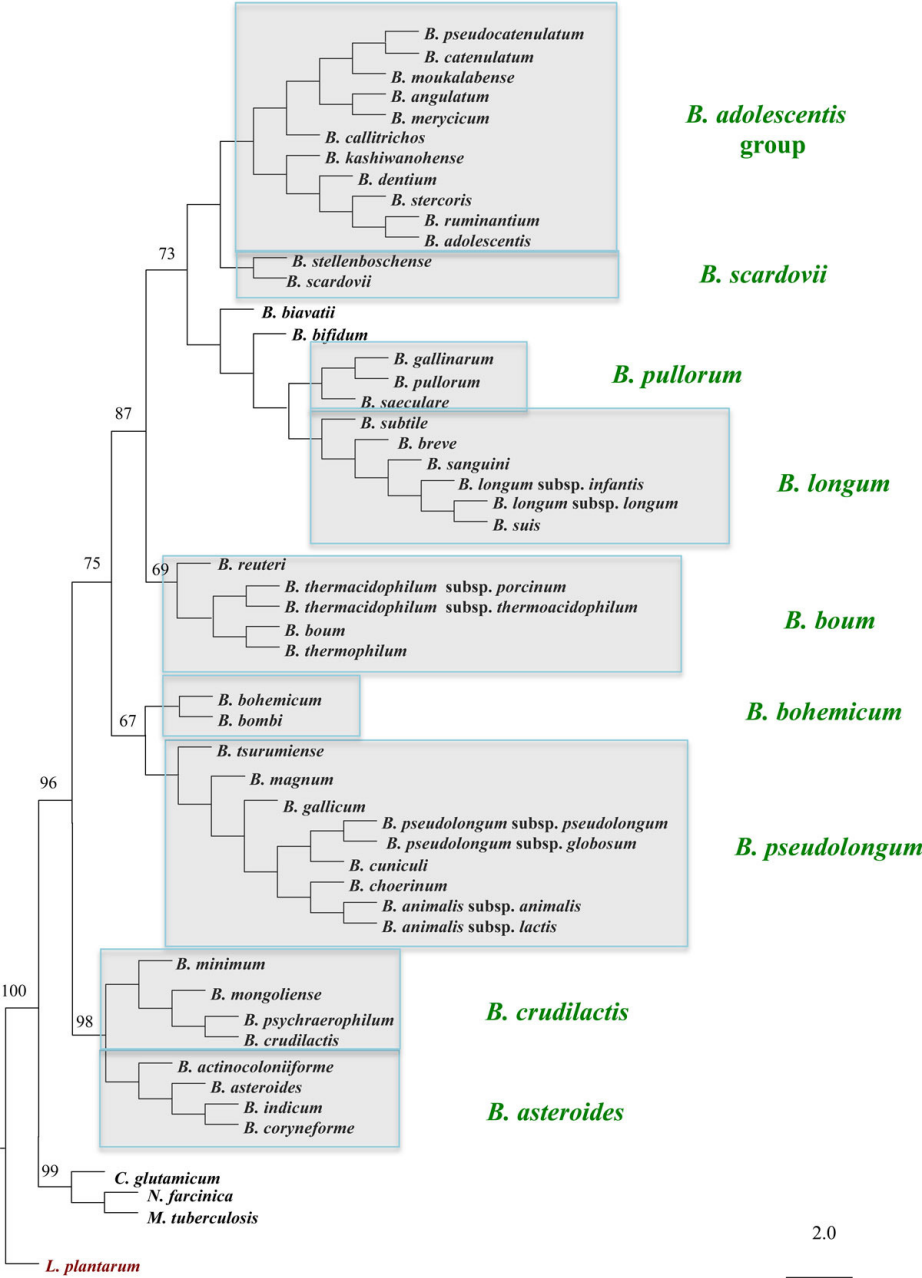
**Rooted neighbour-joining tree based on the alignment (1265 nucleotides) of the 16S rRNA gene (96% of homology) sequence from the 48 so far recognized bifidobacterial taxa (Dec., 2013)**. The phylogenetic groups, as highlighted in grey shaded quadrangles, are the result of a previously performed multilocus analysis [9], integrated with the information resulting from the alignment of the 16S sequence of new bifidobacterial species.

Several of the currently recognized species have only very recently been isolated, such as *Bifidobacterium actinocoloniiforme, Bifidobacterium bohemicum, Bifidobacterium bombi, Bifidobacterium biavatii, Bifidobacterium reuteri, Bifidobacterium callitrichos, Bifidobacterium sanguini, Bifidobacterium stellenboschense, Bifidobacterium stercoris, Bifidobacterium kashiwanohense *and *Bifidobacterium moukalabense *[10-13]. Interestingly, as shown in the 16S rDNA-sequence-based Neighbour-joining tree in Figure 1, three additional phylogenetic clusters seem to be present in *Bifidobacterium*, representing the *B. crudilactis, B. bohemicum *and *B. scardovii *groups. Moreover, microbiota analysis by 16S rRNA-encoding DNA sequences has indicated that more bifidobacterial species are yet to be discovered [4,14], and for this reason we may expect further expansion or refinement of this classification.

Bifidobacteria have been isolated from a variety of ecological niches, such as sewage, fermented milk and anaerobic digestion facilities, yet are most frequently associated with the GIT of humans and animals (in general where the offspring of the bifidobacterial host is raised with parental care which may ensure direct transmission from mother to child/progeny) [15-17].

Their ecological adaptation may also differ among species, some of them for instance can be present in different niches, such as in the case of *Bifidobacterium adolescentis, Bifidobacterium animalis, Bifidobacterium dentium *and *Bifidobacterium catenulatum *(referred to as cosmopolitan bifidobacterial taxa), while others appear to be much more niche-specific, for example *Bifidobacterium cuniculi, Bifidobacterium angulatum *and *Bifidobacterium gallinarum *(so-called specialized bifidobacterial taxa) [18].

### General genome features

Bifidobacterial chromosomes have a genome that ranges in size between 1.9 and 2.8 Mbp, with representatives of the *B. animalis *species possessing the smallest genome, and a representative of *B. longum *subsp. *infantis *harbouring the largest reported genome (Table 2). Bifidobacterial genomes are predicted to encode a substantial number of tRNA molecules, which averages at 52 tRNA-encoding genes per genome, with an outlier in the case of the *B. longum *subsp. *infantis *ATCC 15697 genome, which contains a reported 79 tRNA-encoding genes [19] (Table 2). Interestingly, despite the fact that bifidobacteria possess a tRNA-encoding gene for all common amino acids, genes encoding the amino acyl-tRNA synthetases for glutamine and asparagine are absent in bifidobacteria, and the corresponding charged tRNAs are believed to be produced with the involvement of specific Asn- and Gln-tRNA amidotransferases, performing transamidation of misacetylated Asp-tRNA(Asn) and Glu-tRNA(Gln) [20,21]. The organization of the bifidobacterial chromosome also appears to be consistent with that of a typical bacterial genome, showing a set of conserved genes around the predicted origin of replication (including *rpmH, dnaA, dnaN *and *recF*) and a region rich in AT and multiple DnaA-binding boxes in proximity of the gene specifying the presumed chromosomal initiator of replication (*dnaA*) [22]. Finally, a switch in the direction of the GC skew [the (G-C)/(G+C) value] is observed in bifidobacteria at the origin and terminus of replication, so that the leading strand tends to be higher in Guanine content as compared to its Cytosine content [1,23]. The copy number of rRNA-specifying loci represents another variability in bifidobacterial genomes, and ranges between two and five (Table 2).

**Table 2:** General features of *Bifidobacterium *species with at least one completely sequenced representative.

Organism	Length (Mbp)	ORF content	G+C%	tRNA content	rRNA content	Presence of plasmid	Presence of CRISPR	R/M systems	Presence of prophage	Presence of EPS	Sortase-dependent pili
***Bifidobacterium adolescentis *ATCC 15703**	2.08	1631	59.1	54	5	No	Yes	2	Yes	Yes	1
***Bifidobacterium animalis *subsp. *animalis *ATCC 25527**	1.94	1538	60.4	52	3	No	Yes	1	No	No	0
***Bifidobacterium animalis *subsp. *lactis *DSM 10140**	1.93	1565	60.5	52	4	No	Yes	2	Yes	Yes	1
***Bifidobacterium asteroids *PRL2011**	2.16	1658	60.1	44	2	Yes	Yes	3	No	(Remnant)	0
***Bifidobacterium bifidum *PRL2010**	2.21	1706	62.7	52	3	No	No	7	Yes	No	3
***Bifidobacterium breve *UCC2003**	2.42	1985	58.7	54	2	No	Yes	3	Yes	Yes	3
***Bifidobacterium dentium *Bd1**	2.63	2127	58.5	55	4	No	Yes	2	Yes	No	7
***Bifidobacterium longum *subsp. *infantis *ATCC 15697**	2.83	2416	59.8	79	4	No	No	8	Yes	No	0
***Bifidobacterium longum *subsp. *longum *NCC2705**	2.26	1799	60.3	57	4	Yes	No	6	Yes	Yes	2
***Bifidobacterium thermophilum *RBL67**	2.29	1845	60.1	47	4	No	No	0	Yes	No	2

*In silico *gene predictions show that representatives of the *Bifidobacterium *genus contain an average of 2012 open reading frames (ORFs) per genome, where genomes of *B. animalis *subsp. *lactis *and *B. longum *subsp. *infantis *possess the smallest and largest number of ORFs, respectively, being consistent with their genome size. It is also possible that the lower number of genes observed in *B. animalis *subsp. *lactis *genomes have been caused by their wide-spread utilization in commercial preparations, leading to phenomena of gene loss and genome decay as a result of the adaptation to a nutrient-rich environment, being in agreement with what was also observed in certain lactic acid bacteria [24-26].

### Comparative genomics of bifidobacteria

Comparative genomic analysis of the genus *Bifidobacterium *involving genome sequences of nine bifidobacterial species, *B. longum *subsp. *longum, B. longum *subsp. *infantis, B. adolescentis, B. dentium, B. bifidum, B. animalis *subsp. *lactis, B. angulatum, B. catenulatum *and *B. gallicum *and full nucleotide sequence alignment revealed that these genomes are not colinear, showing a frequent interruption of chromosomal synteny, thereby inferring the existence of significant genome diversity within members of the genus *Bifidobacterium *caused by chromosomal rearrangment events. The *Bifidobacterium *core genome was determined to consist of 506 orthologues that are shared by all nine bifidobacterial species. Functional annotation established that these core genes primarily encode housekeeping functions, including those dedicated to replication, transcription and translation, as well as genes associated with adaptation to a particular niche environment, e.g. genes associated with carbohydrate metabolism, signal transduction and cell envelope biogenesis [27]. The number of truly unique genes (present in a single reference genome but absent in all other analysed genomes), varies between 21 and 230 genes in the nine genomes analysed. The majority of such unique genes have no functional annotation, thereby suggesting that these genes encode for novel biosynthetic or bifidobacterial-host interaction molecules.

Our recent analysis of the pan-genome of the *B. breve *taxon, adopting complete genome sequences of eight *B. breve *strains, namely *B. breve *UCC2003, *B. breve *NCFB 2258, *B. breve *ATCC 20213, *B. breve *JCM 7017, *B. breve *JCM 7019, *B. breve *ACS-071-V-Sch8b, *B. breve *12L, *B. breve *S27 established that these genomes are highly syntenic, with the exception of *B. breve *ACS-071-V-Sch8b and *B. breve *JCM 7017 that harbour inversions in their genomes of 1.1 Mb and 126 Kb, respectively. Analysis of the core and dispensable genome highlighted that the *B. breve *coregenome is comprised of 1,323 core gene families and as expected these encode functions dedicated to cellular housekeeping. From the eight *B. breve *genomes 924 families of variable genes were identified and of these 426 could be classified as truly unique genes. The variable genes encoded proteins involved in capsular polysaccharide biosynthesis, phage resistance (restriction-modification systems and CRISPR loci), production of sortase dependent pili, and carbohydrate transport and utilisation. An extension of the pangenome analysis to include genomes of 6 publically available *B. longum *subsp. *longum *sequences established that 564 gene families were uniquely present in *B. breve *and absent in *B. longum *subsp. *longum*, and while 50% of these encoded hypothetical functions the other 50% were found to encode *B. breve *specific glycosyl hydrolases, ABC transporters, CRISPR genes and mobile elements [28].

### Mobilome

The pool of mobile elements so far found in bifidobacteria is represented by insertion sequences, prophage-like elements and plasmids [29,30]. Regarding insertion sequences, representatives of the main families that have been reported to be present in bifidobacterial genomes are *IS*3, *IS*21, *IS*30, *IS*110, *IS*150, *IS*256, *IS*607/200 and *ISL*3, where in general representatives of *IS*30 appear to be the most abundant and active in bifidobacteria. In fact, it has been suggested that *IS*30 is active in *B. longum *subsp. *longum *and involved in causing genome deletions and rearrangements, supporting the suggested role of IS elements in environmental adaptation of bifidobacteria in general [29].

In contrast to what was initially thought, bifidobacteria appear to be subject to phage infections [30]. Sgorbati et al [31] assessed fourteen strains of *B. longum *by UV and mitomycin C induction for the release of prophage. Bacteriophage were identified from four of the *B. longum *cultures, with the phage head diameters ranging from 49-56 nm while their tails ranged in length from 76-268nm. In fact, the presence of one or more prophage-like elements has been observed in several bifidobacterial genomes (Table 2) [32]. The notion that prophage-like elements may act as a transmission vehicle of genes that do not appear to be essential for phage functions (e.g., hypothetical proteins and glycosyl hydrolase enzymes) is evidence for their potential involvement in lateral gene transfer, and the acquisition of such elements may result in an enhancement of ecological fitness in the receiving members populating the same ecologica niche [33]. Interestingly, bifidobacterial prophage insertion has in several cases been shown to occur at a 35-bp sequence located at the end of the gene specifying tRNA^Met ^[30]. Moreover, an evolutionary analysis conducted on suspected bifidobacterial prophage sequences revealed in a number of cases a high level of sequence identity with prophages present in high and low G+C content Gram-positive bacteria, supporting the hypothesis of phage-mediated DNA exchange between *Actinobacteria *and *Firmicutes*, perhaps facilitated by the fact that these bacteria in certain cases share a common niche [32].

The majority of bifidobacterial strains do not harbour any plasmid and, if they do, a given isolate rarely contains more than one such extrachromosomal genetic element, which then range in size from 1.5-15 Kb. So far, *B. longum *subsp. *longum *and *B. breve *represent the species with the highest number of strains in which plasmids have been identified, with 18 reported plasmid-harbouring representatives of *B. longum *subsp. *longum *and three of *B. breve *[29]. Analysis of their replication (Rep) proteins has indicated that the majority of identified bifidobacterial plasmids replicate by means of the so-called rolling circle mechanism (RCR), while other functions, if encoded, still remain to a large degree unknown [29].

### CRISPR loci and restriction modification systems

As mentioned above, bifidobacteria were until relatively recently not thought to be prone to phage infections since their was no evidence of infection, however, genome analysis of several bifidobacterial strains revealed the presence of predicted phage resistance systems, in particular CRISPR and restriction modification (R-M) systems. The former anti-phage system was not only found in *B. animalis *subsp. *lactis*, but also in one or more representatives of *B. animalis *subsp. *animalis, B. longum *subsp. *longum, B. breve, B. bifidum, B. dentium, B. adolescentis, B. asteroides, B. angulatum *and *B. catenulatum *species [21,29].

Restriction/modification (R-M) systems are ubiquitous among prokaryotes and generally comprise of a restriction endonuclease (REase) and cognate methyltransferase (MTase) [34]. R-M systems serve primarily as defensive instruments that protect prokaryotic cells against invading DNA such as promiscuous plasmids or infecting bacteriophage as the unmodified incoming DNA is targeted by the REase component of the R-M. The host DNA is resistant to cleavage as the recognition sites of the endonuclease are modified by the cognate methyltransferase at adenosyl or cytosyl residues. R-M systems are classified into four groups (designated type I, II, III and IV) on the basis of their subunit composition, co-factor requirement, recognition sequence structure and the cleavage site relative to the recognition sequence [35]. REase activity in *Bifidobacterium *was first described by Khosaka et al. [36,37], who identified the restriction endonucleases BbeI from *B. breve *YIT4006, and BinSI and BinSII from *B. longum *subsp. *infantis *S76e. Subsequently, REase activity was demonstrated in strains of *B. adolescentis, B. bifidum, B. lactentis *(subsequently reclassified as *B. longum *subsp. *infantis*) and *B. longum *subsp. *longum *[38-40]. *In silico *analysis of sequenced bifidobacterial genomes shows that the number of R-M systems varies not only between bifidobacterial species, but also between strains of a particular species (Table 2). The genome of *B. thermophilum *lacks R-M systems, while strains of *B. bifidum *harbour the genetic determinants to encode between four and seven R-M systems and genes encoding up to eight R-M systems have been identified on the sequenced genomes of strains of *B. longum *subsp. *infantis*. The ability to circumvent the R-M complement of strains of *B. longum *and *B. adolescentis *has allowed with the introduction of *E. coli*-bifidobacterial shuttle vectors into these strains by electroporation [41,42], while for *B. breve *strains it has allowed the creation of insertion mutants via site specific homologous recombination or transposon mutagenesis [43], thereby advancing our understanding of bifidobacterial genomics, physiology and metabolism [28,44,45].

### Evidence of horizontal gene transfer

Bacterial genome evolution occurs through various mechanisms, which include gene duplication, chromosomal rearrangements, vertical DNA exchange and intra-species horizontal gene transfer (HGT), events that may facilitate rapid environmental adaptation [46]. For this reason, defining the precise evolutionary distance between bacterial taxa is a complex and difficult task, and the presence of a common ancestor may not be sufficient in measuring the true distance between two phylogenetic groups, when also taking the presence of common functions for genetic adaptation to a common niche into account.

HGT appears to play an important role in increasing the competitiveness of bacteria in their ecological niche, and in bifidobacteria DNA regions acquired through HGT are frequently present in clusters that are randomly dispersed across the genome and in many cases being flanked by mobile elements [21,47,48].

## Intestinal function

### Adaptation to the gastrointestinal environment

In order to survive gastrointestinal transit and transiently colonise the GIT, bifidobacteria must be able to counter the adverse conditions of the oral cavity, stomach and small intestine. In particular, exposure to oxygen or other oxygen-derived free radicals, organic acids, and bile, as well as osmotic stress can have a negative impact on bifidobacterial cell viability and consequently probiotic functionality. Bifidobacteria cope with these stressful conditions by adopting a repertoire of molecular chaperones, bile efflux transporters, bile salt hydrolases, two-component systems and ATPases [49-55]. Tight control of these stress-induced coping mechanisms allows bifidobacteria to rapidly react to various and sometimes complex environmental challenges. This regulation is governed by an interactive nextwork of regulators that include ClgR, HspR, HrcA, and LexA. The regulons are highly conserved among sequenced bifidobacterial genomes suggesting that a universal system for adaptation to osmotic, oxidative and acid stress exists among all members of the *Bifidobacterium *genus [55]. Bile tolerance is among the most crucial properties for a probiotic bacterium as it determines a strain's ability to survive transit through the small intestine. The active extrusion of bile acids and salts that accumulate in the cytoplasm through efflux pumps is a commom mechanism employed by bacteria to counter bile toxicity. Multidrug transporters belonging to the ATP-binding cassette or major facilitator family have been described as mediating bile tolerance in strains of *B. longum *subsp. *longum *and *B. breve *UCC2003 [49,56,57]. In *B. breve *UCC2003 Bbr_0838 encoding a multidrug transporter of the major facilitator family is strongly induced during exposure to bile. Inactivation of Bbr_0838 through insertional mutagenesis rendered the mutant strain, UCC2003-838, more sensitive to cholic acid compared to the parent strain demonstrating that cholate is the main bile component detoxified by Bbr_0838 [49]. Survival in the highly complex and competitive environment of the GIT requires that commensal bacteria including bifidobacteria can protect themselves against host proteases. Some bifidobacterial species achieve protection against human proteases, such as α-antitrypsin and human neutrophil elastase, by a serine protease inhibitor (Serpin), the production of which is regulated by an environmental sensing two component regulatory system [51,53].

### Interaction with the host environment

The mechanisms by which bifidobacteria interact within the gut environment and adhere to the host surfaces are still under investigation and the recent availability of several bifidobacterial genome sequences underlined the presence of a number of macromolecules associated with the cellular envelope and involved in host-microbe interactions [48,58,59]. The capsular or surface exopolysaccharide (sEPS) is one such macromolecule which was shown to contribute to host colonization and persistence by facilitating bifidobacterial long-term colonization of host cells [1,60,61]. Another extracellular structure which is crucial in the process of colonization of the intestinal mucosa is represented by appendices called fimbriae or pili, found in both enteric and oral bifidobacteria [21,62-64]. They can be involved in the establishment of host cell contact and adhesion to the epithelial cells, in cellular aggregation or in biofilm formation, but they may also stimulate a response by the host immune system [48,59]. Bifidobacteria have long been recognized for their ability to prevent pathogen infection, however, the precise mechanism has remained elusive. Recent research has demonstrated that gut pathogen protection conferred by bifidobacteria is associated with LuxS and AI-2 production via a mechanism that may be correlated with iron acquisition (Christiaen et al., submitted).

### Exopolysaccharide production

A bacterial sEPS usually consists of a complex extracellular structure composed of a repetition of mono/oligosaccharides linked through glycosidic bonds, which determines the properties of the homo/heteropolymeric structure. The bacterial sEPS was first studied and characterized in pathogens, where such macromolecules play a crucial role as a virulence factor in the interaction of the bacterium with its host through modulation of the immune system [65].

Differently from pathogens, the sEPS in commensal bacteria (such as bifidobacteria) has only recently received scientific attention and very little is known about its precise biological role. The involvement of sEPS in providing tolerance to bile salts and low pH has previously been eluded to [66], while a recent study conducted in *B. breve *UCC2003 demonstrated that its surface-located EPS promotes *in vivo *persistence by mediating evasion of the B cell-mediated adaptive immune response in the murine gut and preventing the production of proinflammatory cytokines such as IFN-γ, TNF-α and IL-12 [67].

The genome of *B. breve *UCC2003 contains two predicted EPS-encoding gene clusters: *eps1*, elements of which are similar to a cluster involved in the formation of a cell wall-associated phospho-polysaccharide or pellicle in *L. lactis *subsp. *cremoris *MG1363 [68] and *eps2*, which is responsible for sEPS production.

**Figure 2:**
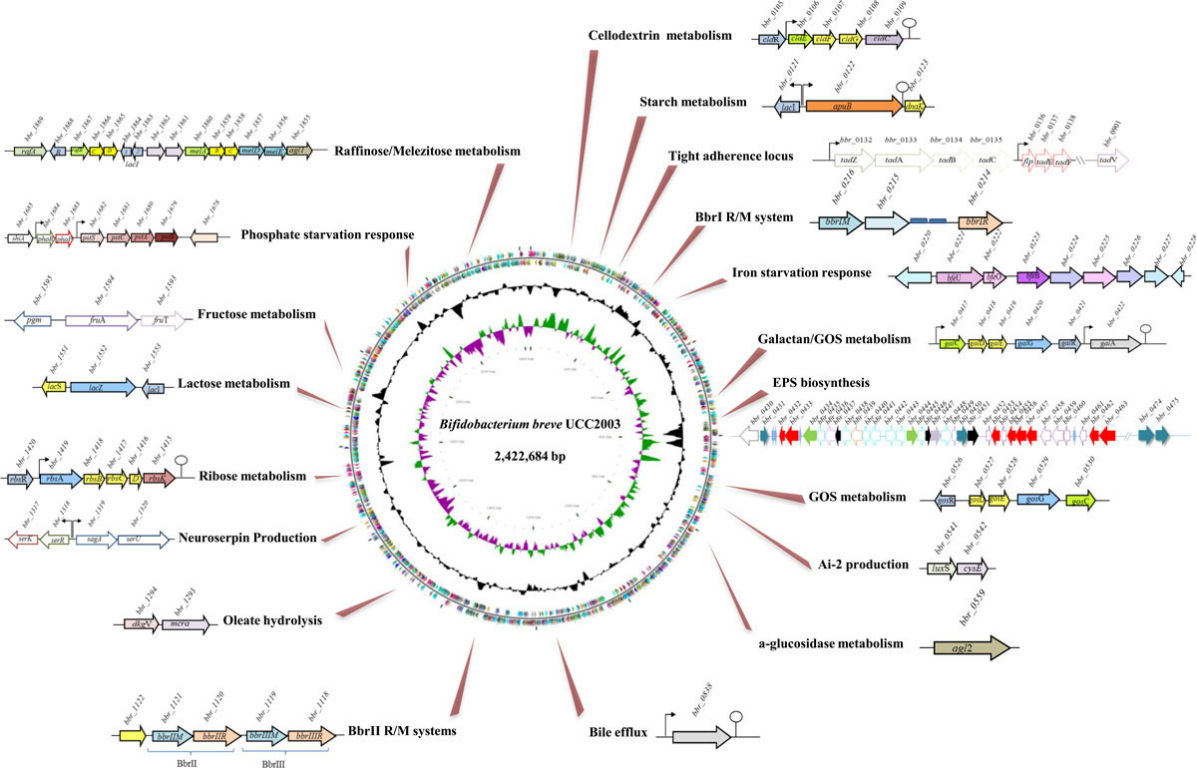
**Genome atlas of *B. breve *UCC2003**. Surrounding the genome are schematic representations of selected genes or gene clusters that have been characterised through comparative and functional genomic approaches using *B. breve *UCC2003 as a model *Bifidobacterium *strain. The proven function of each gene/gene cluster is indicated together with the gene name and gene ID, see text for further explanations and relevant references.

Interestingly, the *eps2 *locus is organized in two adjacent and oppositely oriented operons (called *eps2A *and *eps2B*, Figure 2), one of which is constitutively transcribed, while the other is transcriptionally silent unless a promoter reorientation reverses this situation using a mechanism that is likely to be similar to that observed in *Bacteroides fragilis *[67,69]. More specifically, this promoter inversion is presumed to be catalyzed by the presence of an inverted repeat sequence in the intergenic region between *eps2A *and *eps2B*, and the activity of an as yet unidentified site-specific DNA invertase/recombinase [67]. Recent comparitive analysis on eight fully sequenced *B. breve *genomes revealed the presence of an intact *eps2 *locus in *B. breve *JCM 7017, *B. breve *JCM 7019, *B. breve *689b and *B. breve *S27, while the genomes of *B. breve *ACS-071-V-Sch8b, *B. breve *NCFB 2258 and *B. breve *12L appear to contain only a remnant EPS cluster, where the gene encoding the priming glycosyl transferase is present, yet lacking several genes commonly associated with EPS biosynthesis [28]. Aside from *B. breve*, putative EPS-specifying regions are also present in the genomes of most other bifidobacterial species, for example a complete locus has been identified in members of *B. animalis *subsp. *lactis, B. longum *subsp. *longum *and *B. pseudocatenulatum *[61,68,70] (Table 2). Interestingly, the G+C content of these regions deviates from that of the genome and suggests their acquisition through a HGT mechanism [48]. Further functional analyses are needed to determine the structural diversity of bifidobacterial EPS polymers and their associated biological function.

### Pili in bifidobacteria

The mechanism of interaction between gut microbes and the intestinal mucosa may involve hair-like appendices, named pili or fimbriae, which are exposed on and attached to the cellular surface. Apart from the process of host colonization, pili may also be involved in other cellular functions, which include protein secretion and conjugation [71].

During colonization bifidobacteria are believed to establish an initial contact with the host surface, followed by a more tight and specific adhesion [48]. Two different types of pili are held responsible for this process: the type IVb or so-called tight adherence pili (Tad pili) and the sortase-dependent pili. Both of these extracellular structures are composed of assembled pilin subunits where in the case of Tad pili they are linked by non-covalent interactions and attached to the membrane lipid bilayer, while in the case of sortase-dependent pili they are covalently anchored to the cell wall and their subunit assembly involves the establishment of covalent bonds catalyzed by a dedicated transpeptidase enzyme (so-called sortase) [72].

Tad pili were first characterized in the Gram-negative coccobacillus *Actinobacillus actinomycetemcomitans*, where they are shown to be required for adhesion to host surfaces, colonization and pathogenesis [73,74]. The assembly apparatus is composed of an ATPase (TadA) associated with two membrane proteins (TadB and TadC), together constituting the secretion system, a septum site-determining protein (TadZ), which directs the pilus secretion machinery to a cell-polar position, and finally a peptidase (TadV), which cleaves a leader peptide from the prepilin precursors [75]. This locus is present in both *Bacteria *and *Archea*, and is for this reason termed a **W**idespread **C**olonization **I**sland (WCI) because of its common presence among and apparent mobility between phylogenetically distant micro-organisms [76]. Highly homologous Tad pili-encoding genes are present in all currently sequenced bifidobacterial genomes, which enforces their presumed role in the establishment of a direct interaction with their host. A recent description of the genome sequence of *B. breve *UCC2003, together with an *in vivo *transcriptomic and mutational analysis, confirmed the involvement of Tad pili in murine gut colonization by *B. breve *[48].

In contrast, sortase-dependent pili have been observed decorating the bifidobacterial cell surface and their expression seems to be strongly dependent on growth conditions [64]. One or more sortase-dependent pilus-encoding loci are present in the genome of most, but not all, bifidobacterial species (Table 2), where *B. dentium *represents the bifidobacterial species with the highest number of pilus-encoding loci (Table 2) [21]. A typical sortase-dependent pilus-specifying locus in a bifidobacterial genome consists of a gene cluster composed of one or two pilus subunit-encoding genes with a dedicated sortase-encoding gene placed in an adjacent position [64].

Various sortase-dependent pilus-specifying clusters found in other bacteria are thought to have been acquired through lateral transfer due to their deviating (G+C) content, and a phylogenetic analysis conducted on bifidobacterial gene clusters predicted to be involved in the biosynthesis of sortase-dependent pilus-like structures was shown to be consistent with this notion [71].

Notably, a recent study conducted on *B. bifidum *PRL2010 established an additional function of sortase-dependent pili as they were not only shown to be involved in the specific binding to extracellular matrix components (in this case fibronectin and collagen), but were also shown to be responsible for bacterial aggregation [59]. Perhaps this aggregation phenotype may allow individual cells of this gut commensal to adhere to each other in order to enhance the colonization of the host mucosa, using a mechanism similar to the one described for the probiotic strain *Lactobacillus rhamnosus *GG [77,78]. Moreover, this study demonstrated that such pili play a role in modulating the host immune response, on the one hand inducing a high level of expression of TNF-α cytokines known to be produced at local level as a result of inflammatory disease, while on the other hand acting as low-level inducers of other proinflammatory cytokines (e.g., IL-12), associated with systemic responses [59]. Furthermore, the induction of TNF-α exerted by pili of PRL2010 cells may be crucial for the initiation of cross-talk among immune cells without causing any inflammation or detrimental effects [79]. In fact, the infant's immune system is immature and the presence of pro-inflammatory stimuli such as those provoked by pili of *B. bifidum *PRL2010 may be essential to achieve appropriate developmental immunological programming [59].

### AI-2 production by bifidobacteria

A wide range of Gram-negative and Gram-positive bacterial species produce the quorum sensing molecule AI-2 and for this reason it is often referred to as the interspecies signaling molecule. The key enzyme for AI-2 production is LuxS, which is an essential part of the activated methyl cycle, involved in recycling S-adenosylhomocysteine. More specifically, LuxS catalyzes the cleavage of S-ribosyl-homocysteine to homocysteine and 4,5-dihydroxy-2,3-pentanedione (DPD), which subsequently leads to the production of AI-2 [80]. Although AI-2 is commonly linked to virulence and pathogenicity [81,82], it has recently been shown that probiotic *Lactobacillus *strains, including *Lactobacillus acidophilus *NCFM, *Lactobacillus rhamnosus *GG and *Lactobacillus reuteri*, each harbour a functional *luxS *gene and produce AI-2 [83-86]. We recently established that all *Bifidobacterium *strains sequenced to date harbor a *lux*S gene, and our investigations have demonstrated that all tested bifidobacterial strains, representing 11 species of this genus, were capable of producing AI-2. Through insertional inactivation and subsequent complementation experiments we demonstrated that a functional *lux*S gene is necessary for bifidobacterial AI-2 production. In addition, we observed downregulation of genes associated with iron transport in a *lux*S insertion mutant strain, UCC2003-luxS, during *in vitro *growth. Consistent with this result UCC2003-luxS was shown to be more sensitive to various iron chelators, and unable to colonize the murine gastrointestinal tract, while this mutant also conferred less protection against *Salmonella *infection in a *C. elegans *nematode model. Collectively these data demonstrate the importance of LuxS for bifidobacteria to establish as gut commensals, which also includes their beneficial effect pertaining to pathogen protection/exclusion [87]. Furthermore, our results indicate that LuxS activity is involved in iron acquisition, a property that gives *B. breve *UCC2003 a competitive advantage in iron-limited environments such as the gastrointestinal tract.

### Production of bioactive metabolites

Metabolic end products, such as SCFA, vitamins, polyunsaturated fatty acids such as conjugated linoleic acid also contribute to intestinal functionality and probiotic attributes of bifidobacteria. SCFAs are the end products of bifidobacterial fermentation of complex carbohydrates in the GIT, and have been found to be key for human metabolism as they stimulate water and sodium absorption, lower luminal pH and the bioavailability of toxic amines [88]. Recently, it was shown that acetate produced by bifidobacteria could enhance intestinal defence mediated by epithelial cells and thereby protecting the host against infection by *E. coli *O157:H7 [89]. In addition, while bifidobacteria do not produce butyrate as an end product of fermentation, de Vuyst and Leroy have demonstrated the importance of cross-feeding on acetate by butyrate-producing bacteria in the gut [90]. Butyrate is the primary energy source for colonocytes and has attracted much research interest due to the possibility of its use for the prevention of colon cancer [91]. Conjugated linoleic acid (CLA) refers to a mixture of positional and geometric isomers of the essential fatty acid linoleic acid (C18:2, cis-9, cis-12 octadecadienoic acid). CLA has been reported to be produced by some human bacterial isolates from different bacterial groups that include *Lactobacillus, Propionibacterium, Bifidobacterium, Pediococcus, Enterococcus *and *Lactococcus*. Among bifidobacteria, strains of *B. breve *have been show to produce high levels of CLA [92]. CLA has been shown to exert several health benefits and has been demonstrated to have potent anti-inflammatory, immunomodulatory, anti-obese and anti-carcinogenic activity, along with the ability to improve biomarkers of cardio-vascular health [93].

### Carbohydrate transport and metabolism by bifidobacteria

One way by which gut commensals exert their beneficial effect on their hosts is by degrading diet-derived carbohydrates that cannot be digested by host enzymes, such as plant-derived glycans (e.g., glucans, fructans, xylans, resistant starch, pectins, cellulose, arabinoxylan, and their respective oligosaccharide degradation products), and host glycans (e.g., **H**uman **M**ilk **O**ligosaccharides or HMO, and mucin-type *O*-and *N*-glycans).

Of such carbohydrates, bifidobacteria can degrade certain polysaccharides by extracellular enzymes into mono- and/or oligosaccharides, which are then internalized using mostly sugar-specific ATP-binding cassette (ABC) transporters, permeases, proton symporters and, in a few cases, phosphoenolpyruvate-phosphotransferase (PEP-PTS) systems [21,94]. Once internalized in the cytoplasm, carbohydrates may be subjected to further hydrolysis, epimerization, deacetylation, deamination and/or phosphorylation involving the participation of specific enzymes, such as glycosyl hydrolases, sugar phosphorylases, epimerases, mutases and/or kinases [94].

All necessary genes involved in the utilization of a given sugar are frequently organized in gene clusters containing genes that encode one or more specific glycosyl hydrolases and transport systems, and are usually placed under the transcriptional control of a LacI-type regulator specified by a gene that is also located adjacent to or within such a gene cluster [62].

The fermentation pathway of simple and complex carbohydrates employed by bifidobacteria converges to a specific metabolic route called "bifid shunt" which yields 2.5 ATP molecules per 1 Mol of glucose, 1.5 Mol of acetate and 1 Mol of lactate. The central enzyme of this pathway is represented by the fructose-6-phosphoketolase, of which the encoding gene is widely used as genetic marker for the genus *Bifidobacterium *[95].

In general, glucose and fructose can enter directly into the "bifid shunt" pathway, while other sugars are degraded by the intervention of additional glycosyl hydrolases, depending on the strategy of niche adaptation and carbon source availability [93,96-102].

According to the Cazy database classification (http://afmb.cnrs-mrs.fr/CAZY/index. html) the glycosyl hydrolases that are most commonly found in bifidobacteria belong to the GH13 (α-glucosidase and sucrose phosphorylase), GH36 (α-galactosidase) and GH2/42 (β-galactosidase). Representatives of the GH13 family are typically enzymes responsible for the degradation of substrates with α-glucopyranose units, such as pullulan, glycogen, maltodextrin, starch, and amylopectin, and their presence has been pointed out as a characteristic feature of *B. breve *[103]. Members of the GH36 family frequently represent enzymes dedicated to the hydrolysis of α-galacto-oligosaccharides present in soymilk and various plants (i.e., melibiose, raffinose, stachyose) [102,104].

Enzymes which fall into the β-galactosidase group generate galactose that enters the central carbon metabolism through the Leloir pathway, which is necessary for bacterial growth on (ga)lactose-containing host-derived substrates such as human milk oligosaccharides (HMO) and mucin.

### Carbohydrate degradation capabilities of *Bifidobacterium *species influence their presence and contribution to microbiota composition

The dominance of bifidobacteria in the (breast-fed) infant gut microbiota has been attributed to the ability of certain bifidobacterial species to consume human milk oligosaccharides (HMOs). Recent fecal microbiota compositional analysis from 11 neonates established that *Actinobacteria *represented the dominant phyla at 88.5% with the *Firmicutes *represented at 11.1% [105]. The most abundant classes in the infant fecal samples was *Bifidobacteriales*, being present at 80.6%, while *Lactobacillales *and *Clostridiales *represented the second and third most abundant classes and being present at 7.2% and 3.1%, respectively. The dominant *Bifidobacterium *species detected in the infant fecal samples were *B. longum *and *B. bifidum *at 56.2% and 10.7%, respectively [105], while it was noted that these two *Bifidobacterium *species were apparently absent in a study that analysed the *Bifidobacterium *composition of the adult gut microbiota [106]. The dominance of *B. longum *and *B. bifidum *in the infant gut microbiota is consistent with their ability to use host-derived oligosaccharides such as mucin and HMO. Mucin-type *O*-glycans are constituents of mucins, which are located in different mucosal sites of the body. The four main core glycan structures are made up of a combination of galactose, *N*-acetylglucosamine, *N*-acetylgalactosamine, fucose and sialic acid, linked through various glycosidic bonds [107]. HMOs are synthesized in the mammary gland and contain glucose, galactose, *N*-acetylglucosamine, fucose and sialic acid linked by at least 12 different glycosidic bonds [108]. The core of both mucin-type *O*-glycans and HMOs is composed of the same building blocks, which can be connected together by various glycosidic links in order to assume a range of structures, whose degradation still involves a similar set of enzymes, among others β-hexosaminidases, β-galactosidases and α-sialidases [95,109]. A case of differential host glycan utilization in bifidobacteria is represented by *B. bifidum *PRL2010 and *B. longum *subsp. *infantis *ATCC 15697, of which the former is able to utilize both mucin-type glycans and HMOs [109], while the latter is only capable of degrading HMO, suggesting that a (partially) divergent strategy of adaptation to the infant gut was applied in either of these bacteria [109]. While not all bifidobacterial species can utilize HMO directly, many can cross-feed on HMO degradation products that are liberated by the action of extracellular glycosyl-hydrolases. These degradation products/cross-feeding substrates may include sialic acid, fucose, lacto-*N*-tetraose (LnT) and lacto-*N*-biose and their consumption by other bifidobacterial species is likely to shape the particular composition of the infant microbiota. This nutrient-based crossfeeding or co-operative resource-sharing allows other bifidobacterial species that do not directly utilize HMO to establish themselves in the infant GIT, and supports the notion that the diet has a definitive impact on the gut microbiota composition.

Previous genomic analyses have described how infant associated bifidobacterial species (e.g. *B. bifidum *and *B. longum *subsp. *infantis*) are genetically adapted to utilize host produced glycans such as mucins and HMO [110], while other bifidobacterial species including *B. breve. B. longum *subsp. *longum *and *B. adolescentis *are adapted to crossfeed on host derived glycans, while they also harbor a repertoire of enzymes dedicated to the metabolism of dietary plant-derived oligo- and poly-saccharides. This diversity in carbohydrate utilization allows persistence of particular *Bifidobacterium *species in the microbiota irrespective of host age and host diet. This notion is exemplified by *B. breve *UCC2003 which, despite being a nursling stool isolate, has the capability to utilize several plant derived carbohydrates including starch, galactan and cellodextrins that would comprise part of the adult diet (Figure 2). In addition, *B. breve *strains can efficiently utilize LnT and sialic acid as energy sources and can crossfeed on HMO degradation products allowing this bifidobacterial species to establish as part of the breast fed infant microbiota despite not being capable of directly utilizing HMO (our unpublished results).

## Conclusion

It is just over 10 years since the first *Bifidobacterium *genome sequence was published. Since then there has been an exponential increase in genome sequencing efforts with, in many instances, the goal of identifying the genes, and unravelling molecular mechanisms, associated with a specific probiotic attribute of a particular *Bifidobacterium *strain. Through comparative and functional genomics these efforts have unveiled the mode of action for particular probiotic attributes. The next decades of bifidobacterial research hold great promise and anticipation as additional novel representatives of the *Bifidobacterium *genus are expected to be isolated, while further insights into this intriguing group of bacteria, and the underlying mechanisms that explain how they interact with their human host and impart their probiotic effects will be unveiled.

## Competing interests

The authors declare that they have no competing interests.
